# Genetic heterogeneity correlated with phenotypic variability in 6 Chinese families with Alport syndrome

**DOI:** 10.3389/fgene.2026.1840344

**Published:** 2026-06-05

**Authors:** Jinghan Gao, Huan Zhou, Li Zhang, Zihan Su, Shiguo Liu

**Affiliations:** 1 Department of Medical Genetics, the Affiliated Hospital of Qingdao University, Qingdao, China; 2 Department of Blood Transfusion, Pingyi County People’s Hospital, Linyi, China; 3 Department of Immunology, School of Basic Medicine, Qingdao University, Qingdao, China; 4 Department of Obstetrics, Qingdao Municipal Hospital, Qingdao, China

**Keywords:** Alport syndrome, functional experiment, gene variant, genetic heterogeneity, genotype and phenotype

## Abstract

**Background:**

Alport syndrome (AS) is a common hereditary kidney disease, mainly characterized by hematuria, progressive renal dysfunction, sensorineural hearing loss, and ocular symptoms, which significantly impacts patients the quality of life patients’ quality of life and lifespan. However, due to its atypical and heterogeneous clinical features, the relationship between genotype and phenotype remains complex, posing AS diagnostic challenges.

**Method:**

Genetic variants were screened by whole exome sequencing (WES) followed by verification with Sanger sequencing. Genotype-phenotype analysis was also conducted, and a novel variant (*COL4A3* c.3203G>A) was selected for *in vitro* functional studies.

**Results:**

We identified seven variants in six families, including autosomal dominant (*COL4A3* c.352G>A, *COL4A4* c.71 + 1G>C), autosomal recessive (*COL4A3* c.2736dupA, c.4235G>T), X-linked (*COL4A5* c.512del,*COL4A5* c.3053del), and one spontaneous variant (*COL4A3* c.3203G>A). Functional studies on the novel variants (*COL4A3* c.3203G>A) demonstrated a significantly decrease significant decrease in the mRNA expression level in HEK293 T cells and the weakened cell migration ability.

**Conclusion:**

We identified four novel pathogenic changes causing AS, revealing the genetic heterogeneity of AS and expanding its genotype phenotype spectrum, holding significant implications for prenatal diagnosis.

## 1 Introduction

Alport syndrome (AS), as one of the most common forms of hereditary nephritis, first expounded by Alport (1927), is characterized by persistent hematuria, accelerated kidney function loss, sensorineural hearing loss, and sometimes, ocular abnormalities (Kelly et al., 2017). Most AS patients often onsets as hematuria at early 10 years old, with intermittent or persistent microscopic hematuria aggravated especially after upper respiratory infection, fatigue or pregnancy (Hashimura et al., 2014; Plevová et al., 2017). With disease progression, males exhibit a higher risk of developing end-stage renal disease (ESRD) at a young age, often by 20–30 years, and this is frequently accompanied by sensorineural deafness.

Three distinct *COL4A5* variants were initially identified in three AS families from Utah, comprising a deletion, a Pst I site variation, and an uncharacterized abnormality, which caused nephritis and deafness, with allele-specific severity (Barker et al., 1990). Subsequent research revealed that homozygous or compound heterozygous variants in either *COL4A3* or *COL4A4* were present in AS patients, while their asymptomatic parents (carriers) only had heterozygous variants, thereby establishing these two genes as the pathogenic genes for AS (Mochizuki et al., 1994). All three genetic variants can lead to abnormality of type IV collagen protein, highlighting genetic heterogeneity of AS. As the main extracellular matrix protein in the glomerular basement membrane (GBM), Type IV collagen protein can express in the basement membrane of alveoli, the eye and the cochlea. This widespread distribution enables it to serve crucial structural functions, providing the scaffold structures for cell adhesion and maintaining the normal morphology of the cells (Bhave et al., 2017; Zhang and Ding, 2018).

AS exhibits three primary genetic patterns: X-linked (XL), autosomal recessive (AR) and autosomal dominant (AD), among them, the form of X-linked (XLAS) accounts for 85% (Plevová et al., 2017). Among these, X-linked AS, caused by pathogenic variants in *COL4A5* located on the X chromosome, is the predominant form. *COL4A5* codes α5 chain of type IV collagen and more than 900 kinds of its variants been found including large rearrangement, deletion, insertion, missense variant, nonsense variant and splicing-site splice-site variant (Ma et al., 2011; Liu et al., 2017), distributed throughout the genome with no significant hot spots. *COL4A3* and *COL4A4* code the α3 and α4 chains of type IV collagen respectively and variants in these genes whether dominant or recessive pattern occupied 15%–20% (Mochizuki et al., 1994). Pathogenic variants in these chains mentioned above caused collagen fibrils structural instable, which led to abnormalities of basement membranes such as irregular thickening, thinning or interphase (Bhave et al., 2017).

Although conventional pathological examinations including pathological changes, immunofluorescence and electron microscopy, combined with clinical manifestations, are helpful in diagnosing AS and distinguishing it from thin basement membrane nephropathy (TBMN) (Deltas et al., 2015; Xu et al., 2016), genetic screening of *COL4A3-5* for variants can provide more definitive genetic information given the complexity of genotype-phenotype relationship in AS families (Savige et al., 2022b; Torra et al., 2025). In addition, due to the rapid progression of the disease, the treatment for AS with ESRD is kidney transplantation. While kidney transplantation remains the primary treatment for AS with ESRD, this approach faces challenges including donor scarcity and potential post-transplant anti-glomerular basement membrane nephritis (Temme et al., 2012; Kelly et al., 2017). Notably, comprehensive genetic analysis of *COL4A3-5* variants in family members serves multiple crucial purposes: (1) it enables accurate genetic counseling by identifying inheritance patterns; (2) facilitates early detection of asymptomatic carriers through predictive testing; and (3) provides the basis for prenatal diagnosis - currently the only method for definitive pre-symptomatic identification.

In this study, we collected six cases of patients with AS, conducted the second-generation sequencing to identify the pathogenic variants, simultaneously summarizing and analyzing their clinical characteristics. Furthermore, a novel variant identified in *COL4A3-5* was subjected to comprehensive pathogenicity analysis to reveal its pathogenic mechanism and explain the genetic heterogeneity of AS, which is beneficial for prenatal diagnosis.

## 2 Patient and methods

### 2.1 Patient screening and data collection

From 2018 to 2023, six families were admitted to Qingdao University Affiliated Hospital and met the criteria for hereditary kidney diseases in the study. In accordance with the principle of informed consent, blood samples were collected from the patients themselves and their parents for genetic screening. This work was carried out with the approval of the Ethics Committee of Qingdao University Affiliated Hospital (Approval Number: QYFYWZLL27300). All the patients participating in this study gave their informed consent.

### 2.2 Genetic analysis

Next-generation sequencing (NGS) was performed for comprehensive variant screening to facilitate molecular diagnosis. Next-generation sequencing (NGS) analysis was performed using a targeted panel covering 355 kidney disease-related genes (including *COL4A3*, *COL4A4*, and *COL4A5*). The complete list of genes included in the panel is provided in Supplementary Table S1. Genomic DNA was extracted from 200 μL of peripheral blood using the Qiagen DNA extraction kit (Qiagen, Haan, Germany). PCR amplification was performed using specific primers designed by Primer Premier 5 (Premier Biosoft International, version 5.0) to target all coding exons of 355 genes related to hereditary kidney diseases and their adjacent ±10 base introns. Then, the purified PCR products were subsequently sequenced on the Illumina HiSeq2000 platform with the average sequencing depth of 116.18 × across target regions, and at least with ≥97.97% of target bases covered at ≥20× depth. The raw sequencing data were processed for base reading by the Illumina workflow and stored in FASTQ format (original data). Copy number variations (CNVs) were analyzed using CNV-seq. No pathogenic CNVs were identified in any of the six families. Variant annotation was annotated for their location, type, and conservation prediction using ANNOVAR to determine genomic locations. Variations were screened according to the following criteria: 1) Quality score >30, read depth >10 × , variant depth >5 × , mapping quality >50; 2) Minor allele frequency (MAF) < 0.01 in the 1000 Genomes database and ExAC.Variants with a minor allele frequency (MAF) < 0.01 in the East Asian population of gnomAD v.4.0 were retained for further analysis. To evaluate the pathogenicity of the variations, Polyphen-2, PROVEAN, CADD, and MutationTaster were analyzed as comparison reference databases using the gene library reference gene sequence by ANNOVAR and DNAMAN software version 5.0. In silico prediction tools were selected according to variant type. For missense variants, we used PolyPhen-2, PROVEAN, CADD, and MutationTaster to predict functional impact. For splice-site variants, dedicated splice prediction tools (SpliceAI, MaxEntScan) were employed. ANNOVAR was used for variant annotation, and DNAMAN (v5.0) was used for sequence alignment and analysis where applicable. Variant analysis was performed according to the guidelines for interpreting sequencing variations by the American College of Medical Genetics and Genomics (ACMG) (Richards et al., 2015). The patients and their family members underwent gene sequencing using Sanger sequencing technology.

The Sanger sequencing method was used to verify the *COL4A3-5* variants carried by initially detected by NGS in the patients. Primers were designed to target exonic regions of *COL4A3-5* to amplify. The 20 μL PCR reaction mixture consistently contained: 1.0 U of AmpliTaq Gold DNA polymerase in 1× reaction buffer (20 mM Tris HCl, pH 8.3, 40mM KCl, 2.0 mM MgCl2), 80 ng of template DNA, 20 nM of dNTPs as raw materials, and 0.4 nM of two primer pairs. The amplification process of the target fragment was as follows: first, the template DNA was denatured at 94 °C; then, the template DNA and the primer pairs were re-annealed for 35 cycles: 94 °C for 30 s, 58 °C for 30 s, 72 °C for 30 s; finally, the extension of the chain was carried out at 72 °C for 10 min. A uniform annealing temperature of 58 °C was used for all primer pairs. Next, the purified PCR products were sequenced on the automatic sequencer ABI 3730XL (Applied Biosystems) for variant analysis.

### 2.3 Renal biopsy for proband

After clinical examination and laboratory investigations, an immunohistochemical renal biopsy was performed on the proband. The biopsy samples were processed for multiple staining techniques. Periodic acid-Schiff and Sudan black staining (PASM) demonstrated the glomerular basement membrane and mesangial matrix black, while Masson staining highlighted the glomerular basement membrane, mesangial matrix, and renal interstitium green. Electron microscopy revealed ultrastructural alterations, including characteristic abnormalities in glomerular basement membrane architecture and podocyte foot process effacement, which provided definitive diagnostic information.

### 2.4 Cell culture and transfection

The cell lines present in this study were obtained from Seastar Biotech Co., Ltd., China. HEK293T cells were cultured in a high-glucose DMEM medium at 37 °C with 10% fetal bovine serum. The cells were passaged when they reached 80% confluence. Wild-type and mutant *COL4A3* plasmids were constructed and transfected into HEK293T cells that had reached 70%–80% confluence. Transfection was performed using Lipofectamine-3000 (Thermo Fisher, United States) and the cells were divided into three groups: empty plasmid group (p.EGFP-N1), wild-type plasmid group, and mutant plasmid group (*COL4A3* c.3203G>A). All transfections were performed in triplicate and repeated independently three times.

We selected the *COL4A3* c.3203G>A (p.Gly1068Glu) variant for *in vitro* functional validation. This variant is the only *de novo* variant in our study population, and the proband carrying this variant exhibits relatively severe clinical symptoms, including hematuria and proteinuria, which warrants further mechanistic investigation. Furthermore, this is a missense variant affecting a highly conserved glycine residue within the Gly-X-Y repeat sequence; relying solely on computational modeling tools makes it difficult to accurately predict its precise molecular mechanism. Therefore, functional characterization is necessary to provide independent evidence supporting its pathogenicity. Furthermore, this variant is currently listed in the ClinVar database with a “pathogenicity classification conflict” flag, and no independent validation reports are available. Consequently, we selected this variant for *in vitro* functional validation to provide evidence supporting its pathogenicity.

### 2.5 Real-time fluorescence quantitative PCR

Total RNA was extracted from HEK293T cells that had been transfected for 24 h using the RNA-easy kit (Thermo Fisher, United States). cDNA was generated using a reverse transcription kit (Vazyme, China). The reverse transcription conditions were 42 °C for 2 min, 50 °C for 15 min, and 85 °C for 5 s. The primer sequences were as follows: forward-ACAACGAGAGGCTTTGTCTTCAC and reverse-CTGCCAAGAGTTCCAAGGTCTT. The sample solution was mixed and placed in a real-time fluorescence quantitative PCR instrument for the qPCR experiment. The reaction conditions were 95 °C for 3 min of pre-denaturation, 1 cycle; 95 °C for 15 s of denaturation, 60 °C for 20 s of annealing, 72 °C for 15 s of extension, 40 cycles. The mRNA expression level of the sample measured in the qPCR reaction was calculated using the formula 2-ΔΔCt. The experiment was carried out three times independently, each with technical triplicates.

### 2.6 Transwell cell invasion assay

After 48 h of transfection, the cells were digested with trypsin and collected into a centrifuge tube. The supernatant was removed and the cells were resuspended in serum-free DMEM medium. The lower layer of the chamber was added with DMEM culture medium containing 20% fetal bovine serum, and the suspended cell solution was added to it. It was gently placed in the incubator. Cultured at 37 °C for 12 h. The chamber was taken out, gently washed with PBS, and placed in a 24-well plate. Add 4% polyethylene to the plate for 10 min; wash with PBS again, then place the chamber in 0.1% crystal violet solution for 10 min. Use a cotton swab to wipe away the cells in the cavity and dry it for photography under a microscope. Take three fields of view each, and count the number of migrating cells. Three independent biological replicates were performed for each condition.

### 2.7 CCK8 assay

After cell transfection, place the 96-well culture plate in the incubator to continue culturing for 48 h (at 37 °C and 5% CO_2_). Add 10 µL of CCK-8 solution to each well. Incubate the culture plate in the incubator for 2 h. Measure the absorbance at 450 nm using an enzyme reader. Cell viability was assessed in three separate experiments, each with triplicate wells.

### 2.8 Statistical analysis

The results of the cell function experiments were statistically analyzed using SPSS 26.0 software, and GraphPad Prism6 was used for graphing. A two-independent-sample t-test was employed for statistical analysis to compare the differences between the *COL4A3* variant group and the control group, as well as between the *COL4A3* wild-type plasmid group and the control group. P < 0.05 was considered statistically significant. All experiments were repeated at least three times independently, and data are presented as mean ± SD to ensure the reliability and reproducibility of the statistical results.

## 3 Result

### 3.1 Clinical examination and laboratory tests of the proband

From 2018 to 2023, the clinical manifestations of six patients who met the genetic testing criteria for AS are shown in Table 1. Gene variant-induced AS was identified in the six cases, five of which were pediatric patients. Their clinical manifestation is microscopic hematuria, which is often the earliest and predominant sign, particularly in children, though it can be challenging to differentiate from other renal diseases. The age of onset ranged from 1 year and 1 month to 18 years, with a median age of 4 years and 9 months. The urine analysis indicated that all patients presented with typical hematuria or proteinuria. Female patients generally had milder symptoms than male. Among the four male patients in this study, three presented not only with hematuria and proteinuria but also with extrarenal manifestations, including hypertension, bronchitis, pneumonia and sensorineural deafness. Moreover, with advancing age, poorly controlled hypertension aggravated proteinuria levels. Concurrently, the progression of AS and the severity of related hearing impairment increased with age.

**TABLE 1:** The clinical characteristics of six patients.

Patient	Mutant gene	Gender	Age of onset	Clinical manifestation			Microscopic features
				Hematuresis	Proteinuria	Extrarenal manifestations	
1	*COL4A3*	Female	2	3+	+	/	Mild segmental mesangial proliferation with volume increase and diffuse thinning of the basement membrane in the glomerulus
2	*COL4A3*	Female	7	3+	-	/	Occasionally, the basement membrane is distorted and irregular, the capsule wall is thickened, and protein casts are occasionally observed. There are also mild lesions in the glomeruli
3	*COL4A4*	Male	5	+	+	/	NA
4	*COL4A5*	Male	4	3+	+	Bronchitis hypertension	The basement membrane is uneven in thickness, with the dense layer thickened. Some areas show a torn or spider-web-like appearance, and the foot processes are widely fused. The glomeruli show mild lesions
5	*COL4A3*	Male	1	3+	-	Pneumonia	NA
6	*COL4A5*	Male	18	3+	4+	Sensorineural hearing loss hypertension	Mild mesangial cell proliferation and matrix expansion. The thickness of the Bowman’s capsule increases. Accompanied by tubular hyperplasia, and there is also invasive basement membrane and fibrotic interstitium. The thickness of the GBM varies. Under the electron microscope, division and layering can be observed in the dense layer

We observed that among the three types of genetic variants, all the patients with the X-linked AS caused by pathogenic *COL4A5* variants were male, with the variants inherited maternally. These two XLAS patients presented with hematuria and proteinuria, as well as extrarenal clinical manifestations such as hypertension and deafness. In contrast, the clinical manifestations associated with *COL4A3* and *COL4A4* variants were relatively mild. Notably, biallelic (homozygous or compound heterozygous) variants in the *COL4A3* or *COL4A4* may lead to more severe clinical manifestations compared to heterozygous variants. Patient 5 carried two changes in *COL4A3* (Table 2) and exhibited typical clinical symptoms of hematuria at the age of one. Although the variants originated from both the father and the mother, the parents who carried the variants were phenotypically normal. Although these variations originated from the father and the mother respectively, his 38-year-old parents who carried these variations were phenotypically normal. Among these families, except for patients 1 and 5 whose parents had no abnormalities related to the kidneys, the mothers of the other patients had varying degrees of hematuria, chronic nephritis, and the father of patient 5 had hypertension. Moreover, significant phenotypic heterogeneity was observed even among patients carrying identical pathogenic variants. These phenomena findings indicate high genetic heterogeneity in AS pathogenesis.

**TABLE 2:** Analysis of the pathogenicity of variations.

Patient	Gene	Mutation	Protein consequence	Mutation type	Mutation rating (ACMG)	ACMG codes	Hereditary mode
1	*COL4A3*	c.3203G>A	p. (G1068E)	missense	Uncertain significance	PM2+PP3	AD/AR
2	*COL4A3*	c.352G>A	p. (G118R)	missense	Likely pathogenic	PS1+PM2+PP3	AD
3	*COL4A4*	c.71 + 1G>C	/	splicing	Likely pathogenic	PVS1+PM2	AD
4	*COL4A5*	c.512del	p. (G171VfsTer32)	frameshift	Likely pathogenic	PVS1+PM2	XLD
5	*COL4A3*	c.2736dupA	p. (G913RfsTer27)	frameshift	Likely pathogenic	PVS1+PM2+PP4	AR
		c.4235G>T	p. (G1412V)	missense	Likely pathogenic	PM2+PP3	
6	*COL4A5*	c.3053del	p. (G1018VfsTer3)	frameshift	Likely pathogenic	PVS1+PM2+PP4	XLD

The electron microscopy examination revealed the typical pathological features consistent with AS, including the enlarged glomerular volume of the mesangial area proliferation, and uneven thickness of the GBM. Among the four patients who underwent electron microscopy, patients 4 and patient 6 with *COL4A5* variants showed more severe microscopic manifestations. Specifically, patients 1 and patient 2 showed diffuse thinning of the GBM with mild mesangial proliferation and irregularity of the basement membrane, which is a typical electron microscopic manifestation of TBMN. Patient 4 had uneven thickness of the GBM, thickening of the dense layer, tear-like and spider-web-like changes, and extensive fusion of the foot processes. Patient 6 exhibited the most severe changes, not only including the characteristic changes of AS, splitting and layering of the GBM dense layer, but also presented the signs of chronic and progressive kidney disease, including renal tubular hyperplasia, interstitial fibrosis, and thickening of Bowman’s capsule, and chronic and fibrotic lesions in the kidneys.

### 3.2 Genetic analysis

**FIGURE 1:**
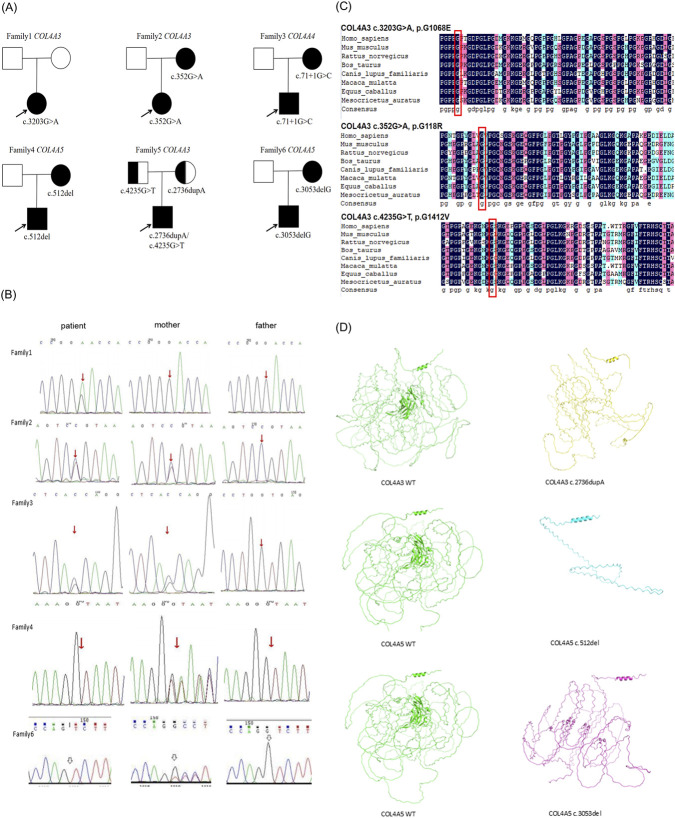
Genetic and bioinformatics analysis. **(A)** Pedigrees of six families. **(B)** Sanger sequencing used to verify the location of gene mutations and the source of the variations. The arrow indicates the position of the mutation. Monoflank indicates homozygous, while biflank indicates heterozygous. **(C)** Protein multiple sequence alignment of *COL4A3* from different species, and the rectangular frame indicates the location of the variant. **(D)** 3D models of the protein before and after mutation.

In summary, seven variants were identified through whole exome sequencing (WES), among which three were novel variants (no record in ClinVar, no record in gnomAD for the East Asian population, and no report in PubMed), including *COL4A3* c.3203G>A (p (G1068E)), COL4A4 c.71 + 1G>C, COL4A5 c.512del (p (G171VfsTer32)), COL4A3 c.2736dupA (p (G913RfsTer27)), and COL4A5 c.3053del (p (G1018VfsTer3)), and four were known variants, including COL4A3 c.3203G>A (p (G1068E)), COL4A3 c.352G>A (p (G118R)), COL4A3 c.4235G>T (p (G1412V)), and *COL4A*5 c.3053del (p.G1018Vfs*3). and *COL4A4* c.71 + 1G>C. Subsequently, we verified that among these six families, two exhibited X-chromosome dominant inheritance, two showed autosomal dominant inheritance, one demonstrated autosomal recessive inheritance, and it is worth noting that there was also a patient with spontaneous variants (Figures 1A,B). All the genetic variats and disease phenotypes in these six families were co-segregated, and these alterations were not found in the normal population database of the same race. these variants were absent from the East Asian population dataset in gnomAD v.4.0. The relevant variations of the subjects in the six families are shown in Table 2.

Among them, family 4 and 6 carry *COL4A5* variants, transmitted by the XLD. In exon 9 of patient 4 *COL4A5 (*NM_000495.5), there was a deletion of guanine at nucleotide position 512 (c.512del), which was a frameshift mutation and was inherited from the mother. This mutation caused the glycine at the 171th codon of the protein to be replaced by valine, resulting in premature termination of translation at the downstream codon 32 (p.Gly171Valfs*32). In patient 4, a hemizygous frameshift variant, *COL4A5* (NM_033380.3):c.512del, was identified in exon 9 and was inherited from the mother. This variant is predicted to result in a premature termination codon (NP_203699.1:p (G171VfsTer32)). According to ACMG prediction, this variation is initially determined to be of uncertain significance. According to the ACMG guidelines, this variant was classified as a likely pathogenic variant based on the evidence codes PVS1 and PM2. While patient 6 carried the c.3053del variant, located on exon 35 of *COL4A5* (NM_033380.3), which was also a frameshift variant, and the variation was inherited from the mother. This frameshift variant resulted in the substitution of valine for glycine acid at codon 1018 of the protein, causing early termination of translation at downstream codon 3 (p (G1018VfsTer3)). According to ACMG prediction, this variation is initially determined to be a likely pathogenic mutation. According to the ACMG guidelines, this variant was classified as a likely pathogenic variant based on the evidence codes PVS1, PM2 and PP4.

Among the patients identified with the *COL4A3* variants (patient1, 2, 5), two were heterozygous, one was a compound heterozygote, and the genetic patterns were all different, indicating a high degree of genetic heterogeneity. All changes described in this study are annotated using the primary transcript of COL4A3 (NM_000091.5, NP_000082.2). Specifically, the c.3203G>A variant in patient 1 is a missense variant located in exon 37. As the only *de novo* variant identified in this cohort, it is predicted to replace the glycine residue at position 1068 with glutamic acid (p.G1068Glu). All variants described in this study are annotated using the primary transcript of *COL4A3* (NM_000091.5, NP_000082.2). Specifically, the c.3203G>A variant in patient 1 is a missense variant located in exon 37. As the only *de novo* variant identified in this cohort, it is predicted to replace the glycine residue at position 1068 with glutamic acid (p (G1068G)). According to ACMG prediction, this variant is preliminarily determined to be a likely pathogenic mutation. According to the ACMG guidelines, this variant was classified as a variant of uncertain significance (VUS) based on the evidence codes PM2 and PP3. Patient 2 carried the c.352G>A variant, located in exon 6 and inherited from her mother, which conforms to autosomal dominant inheritance. This missense variant led to a change in the protein sequence, where the 118th amino acid was replaced by arginine instead of glycine (p (G118R)). According to ACMG prediction, this variant is preliminarily determined to be a likely pathogenic mutation. According to the ACMG guidelines, this variant was classified as a likely pathogenic variant based on the evidence codes PS1, PM2 and PP3. Patient 5 has a compound heterozygous variant in *COL4A3*, with a c.2736dupA variant in exon 33, which is a frameshift variant and inherited from his mother; and a c.4235G>T variant in exon 47, which is a missense variant and inherited from his father. The missense variant caused the amino acid at position 1412 of the protein sequence changing from glycine to valine acid (p.(G1412V)). For the frameshift variant, p.(G913RfsTer27), the glycine at position 913 is changed to arginine, and a termination codon appears at the 27th amino acid after the variant. This case conforms to autosomal recessive inheritance. According to ACMG prediction, both of these variants are preliminarily determined to be likely pathogenic mutations. According to the ACMG guidelines, this variant was classified as a likely pathogenic variant.

In this study, there was only one patient with a *COL4A4* variant. The c.71 + 1G>C variant of patient 3 was located in the second exon of the NM_000092.5 transcript, and it was a splicing variant. The variat originated from his mother, which conforms to autosomal dominant inheritance. According to the ACMG prediction, this variation was preliminarily determined as a likely pathogenic variant, According to the ACMG guidelines, this variant was classified as a likely pathogenic variant based on the evidence codes PVS1 and PM2, as shown in Figures 1A,B.

### 3.3 Bioinformatics analysis

The protein functions of the suspected pathogenic variants were predicted through family analysis and methods, including SIFT, Polyphen-2, PROVEAN, MutationTaster and RDDC. For the three missense variants, all were predicted to be harmful by the protein function prediction software SIFT, PolyPhen2, PROVEAN and MutationTaster, as shown in Table 3.

**TABLE 3:** Pathogenicity prediction of three missense mutations of *COL4A3* variants.

Gene	Nucleotide change	Amino acid change	Exon	SIFT	PolyPhen-2	PROVEAN	MutationTaster
*COL4A3*	c.3203G>A	p. (G1068E)	exon37	0.00	1.00	−6.827	disease causing
*COL4A3*	c.352G>A	p. (G118R)	exon6	0.00	1.00	−7.229	disease causing
*COL4A3*	c.4235G>T	p. (G1412V)	exon47	0.00	1.00	−7.978	disease causing

In addition, all three frameshift variants can lead to the premature appearance of the termination codon. The protein function prediction software Mutation Taster predicts that all are disease-causing variants.

The only splicing variant, c.71 + 1G>C, was predicted by RDDC to be possibly pathogenic with a score of 0.5059. This alteration has two splicing patterns. One is an insertion of 124 bp, replacing the alternative splicing donor, resulting in a frameshift and premature termination; the other is a deletion of 172 bp, causing exon skipping, resulting in a frameshift.

And the conservation of the protein encoded by the genes with missense variants was analyzed using the DNAMAN software. The *COL4A3* gene and protein sequences of different species, including *Homo sapiens*, *Mus musculus*, *Rattus norvegicus*, *Bos taurus*, *Canis lupus* familiaris, *Macaca mulatta*, *Equus caballus* and *Mesocricetus auratus* were obtained from the National Centerfor Biotechnology Information (NCBI) website. Multiple sequence alignments among these species were compared using DNAMAN software, demonstrating that the p.G(1068E), p.(G118R), p.(G1412V) variants were located in a highly conserved *COL4A3* sequence (Figure 1C).

The 3D simulation models of the protein before and after the frameshift variant (*COL4A3*, p.(G913RfsTer27); COL4A5, p.(G171VfsTer32); COL4A5, p.(G1018VfsTer3)) were constructed using SWISS-MODEL, and the amino acid sequences before and after the variant were analyzed using PyMOL 3.0.3 software. The results indicated that significant changes occurred in the amino acid sequence and protein structure after the frameshift variants, accordingly (Figure 1D).

### 3.4 Kidney biopsy

**FIGURE 2:**
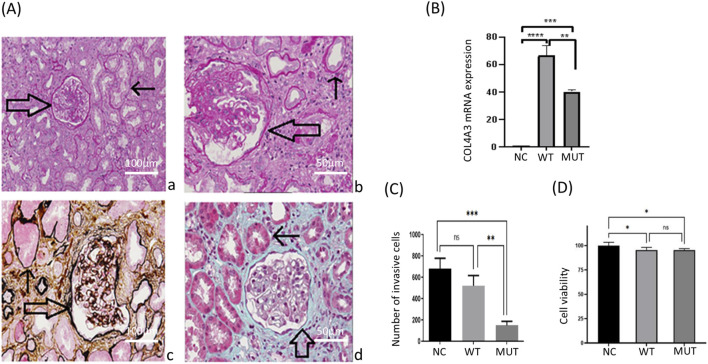
Kidney biopsy and Cell experiment. **(A,a)** HE staining. **(b)** PAS staining: glomerular basement membrane and mesangial matrix appear purplish red. **(c)** PASM staining: GBM and mesangial matrix appear black; Among them, the large arrows represent glomerular mesangial cells and endothelial cells, and the small arrows represent renal tubules. **(d)** Masson staining: GBM, mesangial matrix and renal interstitium appear green. **(B)** The relative expression levels of *COL4A3* mRNA in cells among different transfection groups. **(C)** Transwell assay to investigate the migration ability among different transfection groups. **(D)** CCK8 assay to investigate the proliferation ability among different transfection groups. NC: empty vector group; WT: wild-type plasmid group; MUT: mutant plasmid group. *P < 0.05; **P < 0.01; ***P < 0.001; ****P < 0.0001, ns means no significant difference.

In the process of diagnosis and treatment, in order to clarify the pathological type of patients for the reasonable use of drugs, renal biopsy was performed with the informed consent of patients. Immunohistochemical renal biopsy of the proband 6 showed mild mesangial cellular proliferation matrix expansion and the increased thickness of the Bowman’s capsule wall under light microscopy in Periodic Acid-Schiff staining (PAS) (Figures 2Aa,b). The result of PASM and Masson staining indicated no obvious immune complex deposits in kidney. The pathological changes of the tubule and interstitium were serious. The renal biopsy showed mild acute pathological changes with polynesic atrophy of the renal tubule, accompanied by the incrassate base membrane and the fibrotic interstitium. The interstitium was infiltrated with many inflammatory cells: monocytes, plasmacytes, foam cells and eosinophile granulocyte (Figures 2Ac,d).

### 3.5 RT-qPCR was used to detect the changes in the expression of mutant *COL4A3* mRNA

After 48 h of transfection of 293T cells, qPCR was used to assess the mRNA expression levels of *COL4A3* in each group. The results revealed that compared with the empty vector group (NC), the mRNA expression levels of *COL4A3* in the mutant plasmid group (MUT) and the wild-type plasmid group (WT) were significantly increased (P < 0.01); Compared to the WT group, the expression level of *COL4A3* mRNA in the MUT group with the c.3203G>A variant of the *COL4A3* exhibited significantly decreased (P < 0.01), as illustrated in the Figure 2B.

### 3.6 Transwell and CCK8 assay to investigate the effect of variants on the migration and proliferation ability of HEK293T cells

The effect of the 3203G>A mutant on the cell migration and invasion ability was validated through a transwell invasion assay, revealing that the number of cells crossing the membrane in the MUT group was significantly lower compared to the NC group and the WT group (P < 0.01), as shown in the Figure 2C. This suggests this mutant inhibits the migration and invasion ability of 293T cells.

Furthermore, the CCK-8 experiment results for 293T cells transfected with mutant and wild-type plasmids showed that the proliferation ability of the WT group expressing the *COL4A3* and the MUT group with 3203G>A mutant was significantly lower compared to the NC group (P < 0.05), as shown in Figure 2D. However, there is no significant difference in cell viability between the MUT group and the WT group (P > 0.05). This indicates that overexpression of *COL4A3*, whether mutated or not, reduces cell proliferation, whereas the c.3203G>A variant does not affect cell proliferation.

## 4 Discussion

Secreted by endothelial cells and epithelial cells, type IV collagen constitutes the main extracellular matrix protein in the basement membrane. Its molecules self-organize into a polygonal network, together with laminin networks, collagens, proteoglycans, and some other glycoprotein molecules, forming the core molecular components of the basement membrane. This membrane provides a scaffold for cell attachment and maintains the normal morphology of cells and interacts with adjacent cells to affect cell proliferation, differentiation, adhesion, migration, and molecular filtration (Alport Syndrome Collaborative Group, 2023; Deng et al., 2023). The three α chains of type IV collagen are intertwined to form a triple helix structure. The molecular structure of type IV collagen contains at least six α chains: α1 to α6 chains encoded by six different genes located on different chromosomes. These six α chains spontaneously organize into three prototypical forms, thereby forming three different collagen networks (Mencarelli et al., 2015). Variants in genes such as *COL4A3*, *COL4A4*, or *COL4A5* may lead to a significant reduction or absence of normal *COL4A3-5* protein, resulting in the instability of the mechanical structure of the basement membrane and ultimately leading to renal dysfunction.

This study employed a targeted panel covering 355 genes related to kidney diseases. Although WES has a broader coverage, the advantage of the targeted panel in the clinical diagnosis of AS lies in the following aspects: Firstly, AS is mainly caused by three known genes, and the targeted panel has a higher sequencing depth and coverage in key regions, reducing the risk of omission; Secondly, it is more cost-effective and suitable for areas with limited resources; Thirdly, the coverage of 355 genes still enables the exclusion of other hereditary kidney diseases, strengthening the genotype-phenotype association; Finally, the data analysis is simpler and the turnaround time is shorter, avoiding accidental discoveries. Therefore, the targeted panel is an efficient and practical tool for AS. For cases with a negative panel result but a high clinical suspicion, it is recommended to conduct further WES testing.

In the initial phase of this study, we enrolled 6 patients with AS, including 5 pediatric cases. These patients exhibited atypical clinical manifestations, primarily presenting as microscopic hematuria, which is often difficult to distinguish from other kidney diseases. Due to the hidden nature of the disease, poor prognosis, and variable clinical manifestations, some children may develop ESRD as early as adolescence (Ahmad et al., 2024). AS predominantly affects the renal system, with hematuria being the earliest clinical symptom and most prominent symptom in children. As these patients age, they begin to present other kidney disease symptoms and extrarenal manifestations, such as proteinuria, hypertension, and sensorineural hearing loss (Qian et al., 2025). Due to the rapid progression and poor prognosis of the disease, the current treatment options for it remain limited. However, strict control of fat, protein, calcium, and phosphorus intake in the diet can decelerate the rate of renal failure (Gross et al., 2017). For patients presenting with proteinuria but no renal failure, drug intervention can be attempted: angiotensin-converting enzyme inhibitors (ACEI) may help reduce urinary protein and protect renal function, thereby delaying or preventing the onset and development of ESRD in AS patients (Uchida et al., 2016; Stock et al., 2017). For patients with ESRD, one of the effective treatment options is kidney transplantation. Due to the difficulty in obtaining kidney donors and the occurrence of basement membrane nephritis after transplantation (Johanna et al., 2012), it is particularly important to screen and analyze the *COL4A3-5* variants in the AS family for genetic diagnosis.

This study fully demonstrated the high genetic heterogeneity of AS. In just six patients, we identified three genetic patterns: X-linked inheritance (XLAS, caused by variants in the *COL4A5*), autosomal dominant inheritance (ADAS, caused by heterozygous variants in *COL4A3* or *COL4A4*), and autosomal recessive inheritance (ARAS, caused by compound heterozygous variants in *COL4A3*). Additionally, we discovered one case of spontaneous variant. The diversity of these genetic patterns, as well as the existence of various variant types such as missense and frameshift variants even within the same gene, highlights the complexity of the genetic background of AS. Notably, significant phenotypic differences were observed among individuals carrying the same pathogenic variants, suggesting that in addition to the main pathogenic genes, modifier genes or other environmental factors may also play important roles in disease expression (Ibrahim et al., 2025).

Our research findings are consistent with previous literature reports, indicating that different genotypes are closely related to the severity of the disease. Generally, male patients with XLAS have the most severe symptoms, followed by those with autosomal recessive inheritance, while those with autosomal dominant inheritance have relatively milder symptoms (Kashtan et al., 2018; Puapatanakul and Miner, 2024). In this study, male patients carrying *COL4A5* variants presented with the typical triad of “hematuria, proteinuria, and sensorineural hearing loss”, accompanied by hypertension. With age progresses, proteinuria and hearing loss progressively worsened. In contrast, patients with heterozygous variants in *COL4A3* or *COL4A4* showed milder clinical manifestations, mainly isolated hematuria or mild proteinuria.

Frameshift variants, including *COL4A5* c.3053del, *COL4A5* c.512del, and *COL4A3* c.2736dupA, are associated with early-onset and severe forms of the disease, often progressing to end-stage renal disease (ESRD) before puberty. In our case series, patients 6, 4, and 5, who carried frameshift variants, exhibited relatively more severe symptoms. Frameshift variants result in protein truncation, preventing the synthesis of α3α4α5(IV). The persistent α3α4α5(IV) network in the glomerular basement membrane (GBM) induces biomechanical stress, activates the endothelin-A receptor, and promotes the formation of mesangial foot processes and matrix protein deposition (Caparali et al., 2025). Missense variants, particularly those affecting highly conserved glycine residues in the Gly-X-Y repeat sequence—such as *COL4A3* p.(G1068G) and p.(G1412V)—also result in severe disease but present with more variable clinical manifestations. Glycine is essential for the tight helical arrangement of type IV collagen; replacing it with larger or charged amino acids, such as arginine, valine, or glutamic acid, disrupts the stability of the triple helix. Although α3α4α5(IV) can be synthesized, it folds abnormally, remains trapped in the podocyte endoplasmic reticulum, triggers endoplasmic reticulum stress and the unfolded protein response, and reduces secretion to the GBM (Caparali et al., 2025). The nature of the substituent amino acid may influence disease severity: branched-chain amino acids, such as Val (p.(G1412V)), may cause more severe structural distortions, and symptoms are also relatively severe (Savige et al., 2022a; Gibson et al., 2022). However, these trends require validation through larger-scale cohort studies.

However, our research also revealed some notable details. Unlike the findings in some previous studies where the onset occurred in childhood with relatively slow progression (Jais et al., 2000; Jais et al., 2003; Puapatanakul and Miner, 2024). In this study, one XLAS patient (Patient 6) showed significant chronic pathological changes under the electron microscope, such as renal tubular hyperplasia and interstitial fibrosis, suggesting that the disease progression might be more rapid. Additionally, Patient 5, who is a heterozygous mutant of *COL4A3*, exhibited typical clinical symptoms at the age of one, while the parents carrying the mutant genes demonstrated normal phenotypes. This perfectly illustrates the genetic characteristics of ARAS and emphasizes the importance of conducting double gene variant screening in early-onset, severe cases of children without obvious family history (Mencarelli et al., 2015).

With the widespread use of genetic testing, the role of renal biopsy in AS is changing. Performing WES alone can establish molecular diagnosis, clarify the genetic pattern, and guide family counseling. However, renal biopsy detects ultrastructural changes in the glomerular basement membrane (thinning, thickening, layered arrangement), and through immunostaining of collagen IV α chain, it can confirm the significance of ambiguous variant types; and it can provide prognostic information by evaluating fibrosis and glomerular sclerosis, which is helpful in excluding other glomerular diseases. Therefore, although genetic testing is the preferred method, it is a useful auxiliary means when the genetic test results are unclear, the clinical manifestations are atypical, or prognostic information is needed.

Electron microscopy of renal tissue serves as the gold standard for diagnosing AS. The electron microscopy results of this study have a good correspondence with the genotype and clinical manifestations. Patients carrying the heterozygous variant of *COL4A3* (patients 1 and 2) showed diffuse thinning of the basement membrane in their electron microscopy, which is similar to the pathological features of TBMN, suggesting that it might be a mild phenotype or early stage of AS. While the two XLAS patients (patients 4 and 6) exhibited typical AS characteristic lesions, such as uneven thickness of the basement membrane, layer disintegration of the dense layer, and extensive fusion of foot processes, patient 6 experienced the most severe lesions and had already developed chronic kidney disease-related fibrotic lesions, which provided a pathological basis for its severe clinical manifestations and confirmed that male XLAS patients have a poorer prognosis.

This study identified a total of 7 variants through, including 3 novel variants that had not been reported previously: *COL4A3* c.3203G>A (p.(G1068E)), *COL4A4* c.71+1G>C, *COL4A*5 c.512del (p.(G171Vfs32)), and *COL4A3* c.2736 dupA (p.(G913Rfs27)). *COL4A5* c.512del(p.(G171VfsTer32)), *COL4A3* c.2736dupA (p.(G913RfsTer27)), and *COL4A5* c.3053del (p.(G1018VfsTer3)). A *de novo* variant *COL4A3* c.3202G>A c.3203G>A, whose functional experiments revealed a decrease in mRNA levels and specifically inhibited cell migration ability, provides important experimental evidence for understanding the molecular pathology of this variant leading to AS. The discovery of these new variants has expanded the gene variant spectrum of AS. According to the ACMG guidelines, most of these new changes are predicted to be “likely pathogenic”. In the future, these new variants will need to be further validated for their pathogenicity through functional experiments or through the accumulation of more family case studies, in order to clarify their specific phenotypic associations.

Type IV collagen is the main extracellular matrix protein in the basement membrane and affects cell migration. This description is highly consistent with the results of our Transwell experiment - the *COL4A3* variant significantly weakened the cell migration ability. After collagen IV binds to integrin receptors, it can activate intracellular signal cascades, such as the Rho GTPase pathway, which is crucial for cytoskeleton reorganization and cell migration (Caparali et al., 2025). Additionally, the *COL4A3* cahnge has been proven to cause endoplasmic reticulum stress, which is due to the retention of misfolded collagen chains within the cells. This cellular stress response can indirectly damage various cellular functions, including migration, by activating pro-apoptotic pathways (Pieri et al., 2014; Puapatanakul and Miner, 2024). Therefore, although *COL4A3* is traditionally regarded as a structural protein, its role in cell-matrix signal transduction and intracellular protein homeostasis implies that its variant can affect cell migration. Thus, the impairment of cell migration may have a significant impact on the progression of AS. The migration and motility of podocytes are crucial for maintaining the fine structure of the slit diaphragm and for repairing local damage to the glomerular basement membrane. Therefore, the observed decline in migration ability suggests that the *COL4A3* variant not only weakens the structural integrity of the basement membrane, but may also directly affect the intrinsic function of podocytes, thereby promoting podocyte foot process fusion, cell detachment, and the occurrence of progressive proteinuria.

The results of this study emphasize that for children and adolescents with persistent hematuria, especially those with proteinuria, hearing abnormalities, or a family history, comprehensive genetic testing including NGS should be carried out as early as possible, to achieve early and precise diagnosis and intervention.

## 5 Deficiency and prospect

Although this study has provided preliminary insights into the genotype-phenotype relationship of AS, the following limitations still need to be acknowledged.

We only included six probands from a single center, resulting in a relatively small sample size and limited statistical power. However, this scale reflects the rarity of AS. The incidence of X-linked type is approximately 1:10,000, and that of autosomal recessive type is approximately 1:50,000 (Sun et al., 2023). The strict inclusion criteria and in-depth analysis of genotype-phenotype relationships support the rationality of this study. However, the conclusions still need to be verified by multi-center studies. We will conduct retrospective and prospective multi-center cohort studies in collaboration with multiple domestic hospitals in subsequent research to expand the sample size, include people from different regions, and further verify the genotype-phenotype association patterns discovered in this study and assess their clinical applicability.

In addition, this study utilized HEK293T cells for functional validation, which has certain limitations. HEK293T cells are derived from human embryonic kidneys and are not podocytes; therefore, they cannot fully mimic the podocyte microenvironment of the glomerular basement membrane in AS. We selected HEK293T cells because of their high transfection efficiency, ability to stably express exogenous proteins, and low endogenous *COL4A3* expression, which effectively minimizes interference from wild-type background signals. However, this conclusion still needs to be validated in models that more closely mimic physiological conditions. Future studies plan to use human podocyte cell lines to express this variant, evaluate key phenotypes such as podocyte adhesion, migration, and collagen IV deposition, and further conduct *in vivo* functional experiments in podocyte-specific *COL4A3* knock-in mouse models.

In conclusion, although this study has limitations such as a small sample size and inherent limitations of *in vitro* cell models, through in-depth analysis of rare cases, the genetic-phenotypic association patterns of AS have been preliminarily revealed. In the future, through multi-center collaboration and validation with models closer to physiological conditions, it is expected to provide a more solid theoretical basis for the precise diagnosis and treatment of AS.

## Data Availability

The original contributions presented in the study are publicly available. This data can be found at the ClinVar database under accession numbers SCV007592732, SCV007592755, and SCV007592756.
