# Supplementary material for: Genetic heterogeneity correlated with phenotypic variability in 6 Chinese families with Alport syndrome
Source: Front Genet. 2026 Jun 5;17:1840344. doi: 10.3389/fgene.2026.1840344 (PMC13278684; doi:10.3389/fgene.2026.1840344)
Supplement: Supplementary file 1 [file DataSheet1.docx]

**355 targeted genomic sequences related to kidney diseases**

| *ABCB1* | *ACTN4* | *ALDH3A1* | *ALG1* | *ALG13* | *ANKFY1* | *ANLN* | *APOA1* |
| --- | --- | --- | --- | --- | --- | --- | --- |
| *APOE* | *APOL1* | *APTX* | *ARHGAP24* | *ARHGDIA* | *AVIL* | *B2M* | *BDNF* |
| *BRAF* | *C3* | *CARD8* | *CD151* | *CD2AP* | *CD44* | *CD46* | *CDK20* |
| *CFB* | *CFH* | *CFHR1* | *CFHR3* | *CFHR5* | *CF1* | *CLCN5* | *COL4A3* |
| *COL4A4* | *COL4A5* | *COL4A6* | *COQ10A* | *COQ10B* | *COQ2* | *COQ3* | *COQ4* |
| *COQ5* | *COQ6* | *COQ7* | *COQ8A* | *COQ8B* | *COQ9* | *CRB2* | *CUBN* |
| *DEFA4* | *DGKE* | *DLC1* | *DSTYK* | *E2F3* | *EMP2* | *EPAS1* | *ETFDH* |
| *FAT1* | *FDX2* | *FDXR* | *FGA* | *FLG* | *FN1* | *G6PC1* | *GAPVD1* |
| *GC* | *GLA* | *GSN* | *INF2* | *ITGA3* | *ITGB4* | *ITSN1* | *ITSN2* |
| *KANK1* | *KANK2* | *KANK4* | *KIRREL1* | *LAGE3* | *LAMA5* | *LAMB2* | *LAMB3* |
| *LAMC2* | *LCAT* | *LMNA* | *LMX1B* | *LYZ* | *MAFB* | *MAGI2* | *MEFV* |
| *M1F* | *MMACHC* | *MME* | *MT-TL1* | *MYCT1* | *MYH9* | *MYO1E* | *NARS2* |
| *NEIL1* | *NEK8* | *NEU1* | *NPHP3* | *NPHP4* | *NPHS1* | *NPHS2* | *NUP107* |
| *NUP133* | *NUP160* | *NUP205* | *NUP85* | *NUP93* | *NXF5* | *PCRL* | *OSGEP* |
| *PAX2* | *PAX6* | *PDSS1* | *PDSS2* | *PEX1* | *PLA2R1* | *PLCE1* | *PMM2* |
| *PODXL* | *PTPRO* | *REN* | *SAA1* | *SCARB2* | *SGPL1* | *SLC17A5* | *SLC35A2* |
| *SMARCAL1* | *SPRY2* | *SYNPO* | *TBC1D8B* | *THSD7A* | *TNS2* | *TP53PK* | *TPRKB* |
| *TRPC6* | *TSC2* | *TTC21B* | *TTR* | *UMOD* | *VPS33A* | *WDR4* | *WDR73* |
| *WT1* | *XPO5* | *ZMPSTE24* | *ZNF543* |  |  |  |  |
